# Evaluating clinical response to primary endocrine therapy in elderly breast cancer patients in routine practice

**DOI:** 10.1007/s10549-025-07809-0

**Published:** 2025-09-12

**Authors:** Tessa L. Dinger, José H. Volders, Anne Kuijer, Johannes C. Kelder, Annemiek Doeksen, Emily L. Postma, Thijs van Dalen

**Affiliations:** 1 Department of Surgery, St. Antonius Hospital Utrecht, Utrecht, The Netherlands; 2 Department of Surgery, Diakonessenhuis Utrecht, Utrecht, The Netherlands; 3 Department of Research and Statistical Analysis, St. Antonius Hospital Nieuwegein, Nieuwegein, The Netherlands; 4 Department of Surgery, Erasmus Medical Centre, Rotterdam, The Netherlands

**Keywords:** Elderly, Endocrine therapy, Clinical response, Breast cancer

## Abstract

**Purpose:**

Endocrine therapy (ET) can be used as a definitive treatment in frail and elderly breast cancer patients who are unwilling or deemed unfit to undergo surgical treatment. This study evaluated the clinical response to ET as primary treatment in elderly patients with non-metastatic, oestrogen receptor (ER) positive breast cancer, by evaluating its effectiveness over time.

**Methods:**

Elderly patients (≥ 70 years) with ER-positive breast cancer who had been treated with ET as primary treatment between 2008 and 2015 in two Dutch hospitals were identified through the Netherlands Cancer Registry. The primary outcome was the objectively measured clinical response at various time intervals after initiation of ET.

**Results:**

Out of 122 patients (median age, 86 years), 100 (82%) received ET as definitive treatment, whereas 22 (18%) received ET as neo-adjuvant endocrine therapy. Over the 3-year observation period, 25% of patients had died and 29% underwent invasive local treatment. The overall response rate after 3 years was 14% for all 122 patients and 30% for the 56 patients who were still alive and had not undergone local treatment after 3 years.

**Conclusion:**

The observed clinical response to ET in a consistent proportion of patients over time suggests it may be a viable option for a selection of frail and elderly breast cancer patients with limited life expectancy.

**Supplementary Information:**

The online version contains supplementary material available at 10.1007/s10549-025-07809-0.

## Introduction

In developed countries, the increase in life expectancy has resulted in a greater number of elderly women presenting with breast cancer [1, 2]. Nowadays, approximately, one third of all newly diagnosed breast cancer patients are aged 70 years or over [3]. The biological characteristics of breast cancer in these patients are generally more favourable, with lower-grade tumours and increased oestrogen receptor (ER) expression [4, 5]. Endocrine therapy (ET) is an important component in the multimodal treatment of breast cancer and is usually administered in the adjuvant setting following surgical treatment [6].

In ER-positive elderly breast cancer patients who are unwilling or deemed unfit to undergo surgery, ET can be considered as a primary treatment option [7]. When ET is chosen as definitive primary treatment in elderly patients, eventual tumour progression is accepted and may then require a switch in ET, radiotherapy or salvage surgery [8]. Not providing initial surgical treatment for this group of patients remains controversial, as literature suggests that omission of surgery has an adverse impact on breast cancer-specific survival, deeming ET inferior to surgery [9–11]. Nonetheless, the use of ET in elderly patients has increased in the Netherlands in recent years, reflecting its role as a primary treatment option for a selected group of patients with limited life expectancy [12, 13].

In a previous study, we demonstrated that in patients who were treated with ET, 16% developed uncontrolled local disease after 5 years of follow-up. Additionally, the overall survival rate was 42%, and while 20% of the deceased patients had metastatic or locally progressive breast cancer at the time of death, cardiovascular disease, infectious diseases and old age or dementia were reported as the cause of death in the remaining 80% [14].

In the present study, we aim to evaluate the clinical response of breast tumours to ET over time in elderly breast cancer patients receiving ET as primary treatment. This information may help better inform clinicians and elderly patients with ER-positive breast cancer about what to expect when considering this treatment option.

## Patients and methods

Female patients aged 70 years or older with ER-positive invasive breast cancer, initially treated with ET between 2008 and 2015 in two non-academic Dutch hospitals, were identified through the Netherlands Cancer Registry (NCR). The Committee of Privacy of the NCR approved the use of data for this study. Data on patient, tumour and treatment characteristics at the time of diagnosis were available in the NCR. Patients with T4 disease or distant metastases at initial diagnosis were excluded. The medical records of the identified patients were reviewed and patients who received ET as primary treatment, who were actively monitored and for whom radiological and/or physical evaluations were present in the medical charts during follow-up, were included. Patients who received ET to postpone surgery and were actively monitored until surgery were included, and patients who started ET but did not undergo subsequent response assessments were excluded.

The NCR provided the following clinicopathologic characteristics: age at diagnosis, clinical tumour size, axillary lymph node status, histologic tumour subtype, unifocal or multifocal presence, Bloom and Richardson malignancy grade, oestrogen and progesterone receptor status and human epidermal growth factor receptor 2 (HER2) expression. The clinical records of all patients were retrospectively reviewed to collect information regarding the prescribed ET regimen as well as regarding the patients’ and physicians’ considerations to opt for ET as well as its intention: as a definitive treatment or as a means to postpone intended surgery using neo-adjuvant endocrine therapy (NET).

Follow-up commenced at the time of diagnosis and ended on January 1 st, 2022. There was no formal protocol for the follow-up of patients who received ET, but these patients were usually seen in the outpatient clinic with 3- to 6-month intervals in the first year and less frequently thereafter. Ultrasonography was the preferred imaging technique to monitor tumour size, and physical and radiological examinations were usually alternated. During the follow-up period, data on the measured size of the primary tumour were collected for patients receiving ET by reviewing the radiology reports and descriptions of physical examination in medical records. Information on invasive local treatment (radiotherapy or surgery), the development of metastases and death were also obtained from the medical records, and additional information about vital status was provided by the NCR through linkage with the Municipal Personal Records Database.

The primary outcome of the study was the measured tumour response at consecutive time intervals following initiation of ET in patients who were actively treated: 0–6, 6–12, 12–18 and 18–24 months and 2–3 years. For each time interval, tumour response was determined in patients who were on active treatment and visited the outpatient department and in whom tumour size measurements were obtained during the time interval of interest. Response to treatment was assessed by comparing the maximum tumour size during the follow-up period of interest with the corresponding baseline measurement and categorized in accordance with the Response Evaluation Criteria for Solid Tumours (RECIST) [15] cut-off values to document complete remission (CR; disappearance of the target lesion), partial remission (PR; ≥ 30% decrease of target lesion), progressive disease (PD; ≥ 20% increase of target lesion) and stable disease (SD; < 20% increase and < 30% decrease of target lesion). The overall response rate was the sum of the proportions of patients with a partial or complete response and was reported for the patients in the last observation period.

### Statistical considerations

The distribution of categorical baseline characteristics is presented as percentages and continuous variables as median and interquartile range (IQR). Kaplan–Meier methodology was used to assess cumulative overall survival at 3 and 5 years for the entire cohort, with a 95% confidence interval (95% CI), while the cumulative risk of undergoing invasive local treatment was determined in patients who received ET as a definitive treatment, i.e. excluding patients who received ET as NET, using the Fine-Gray sub-distribution hazards model.

For each observation period, the distribution of observed tumour responses in patients who underwent tumour size assessment, as well as the absence of size assessments in patients for whom no measurements were available due to not visiting the hospital or not undergoing measurements, was presented as the actual proportions of all patients. These actual proportions for the observation intervals were presented together with the cumulative proportions of patients who had undergone planned surgery, salvage local treatment or who had died from the time of diagnosis until that observation period. Statistical analysis was performed using R, version 4.3.1 (R Foundation for Statistical Computing, Vienna, Austria).

## Results

**Fig. 1:**
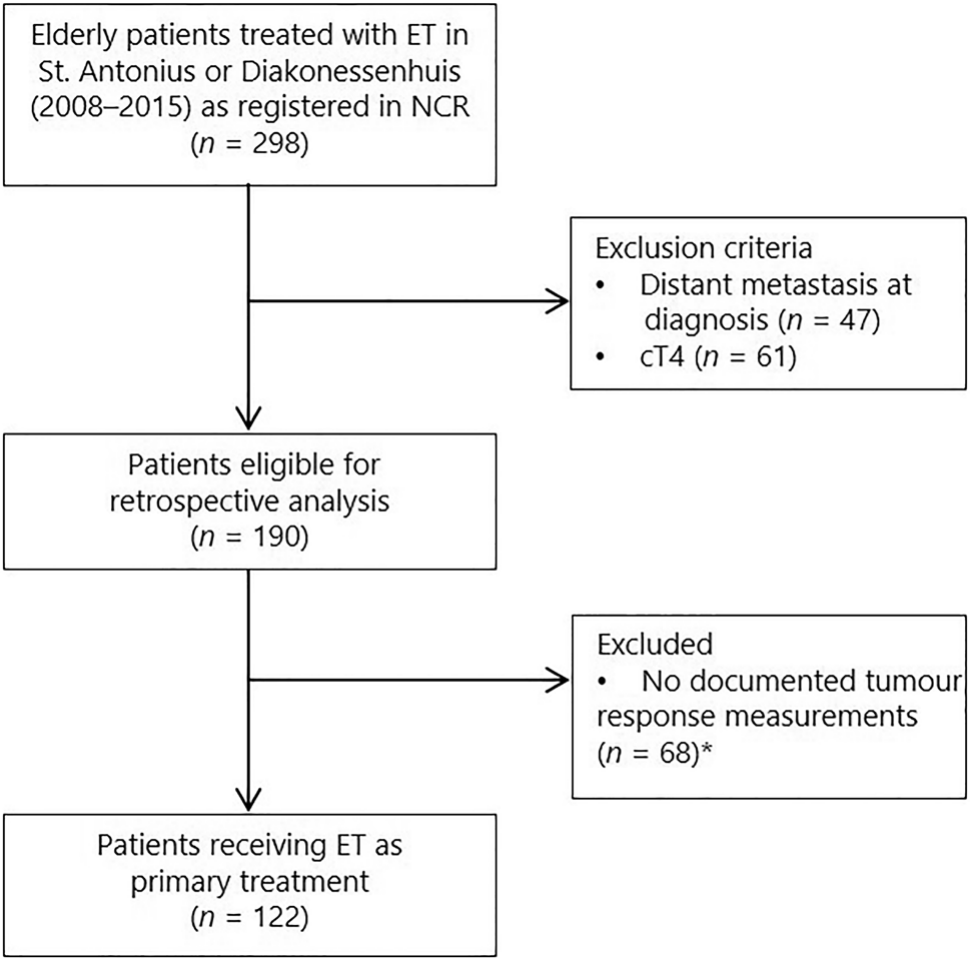
Flowchart of patient selection from the Netherlands Cancer Registry. *NCR* Netherlands Cancer Registry, *ET* endocrine therapy, *cT* clinical tumour stage. * Patients were excluded due to the absence of subsequent tumour response measurements after the initial diagnosis, caused by non-compliance to follow-up appointments

A total of 298 elderly female patients with ER-positive invasive breast cancer who received ET as primary treatment were identified through the NCR. Exclusion of patients with distant metastases (*n* = 47) or with a cT4 tumour at diagnosis (*n* = 61) resulted in 190 eligible patients for analysis (Fig. 1). After reviewing the medical records, another 68 patients were excluded due to the absence of clinical response measurements or any meaningful clinical information in their charts during follow-up. Most of these patients had been referred to their primary care or nursing home physicians after starting ET and received no active follow-up.

The study population consisted of 122 patients. Baseline characteristics are presented in Table 1. The median age at diagnosis was 86 years (IQR, 81–89 years), and 69% of patients had ≥ 4 comorbidities at diagnosis. Patients were predominantly diagnosed with cT1-2 tumours and without clinical lymph node involvement. ET was administered as a definitive treatment to 100 patients, with patient preference being the primary reason for ET (44%), whereas 22 patients received ET as NET. An aromatase inhibitor was given as primary ET in 91% patients.

**Table 1:** Patient, tumour and treatment characteristics

	No. of patients (n = 122)	
*Patient characteristics*		
Median age in years (IQR)	86	(81–89)
No. of comorbidities		
0	7	(5.7)
1	9	(7.4)
2	7	(5.7)
3	15	(12.3)
≥ 4	84	(68.9)
No. of medications^a^		
0–2	23	(18.9)
3–4	28	(23.0)
≥ 5	71	(58.2)
*Tumour characteristics*		
Median tumour size at diagnosis in mm (IQR)	22	(17–32)
Clinical T-stage		
T1	48	(41.4)
T2	62	(53.4)
T3	6	(5.2)
* Missing*	*6*	
Clinical N-stage^b^		
N0	94	(84.7)
N1	10	(9.0)
N2	7	(6.3)
* Missing*	*11*	
PR status		
Positive	90	(77.6)
Negative	26	(22.4)
* Missing*	*6*	
Her2 status		
Positive	6	(7.4)
Negative	75	(92.6)
* Missing*	*41*	
Bloom & Richardson score		
I	7	(22.6)
II	17	(54.8)
III	7	(22.6)
* Missing*	*91*	
Histological subtypes		
IDC	96	(82.8)
ILC	15	(12.9)
Other	5	(4.3)
* Missing*	*6*	
Multifocality		
Yes	12	(11.1)
No	96	(88.9)
* Missing*	*14*	
*Treatment characteristics*		
Reason for ET		
Neo-adjuvant endocrine therapy	22	(18.0)
Definitive treatment	100	(82.0)
Age	37	(37.0)
Comorbidities or unfit for surgery	19	(19.0)
Patient preference	44	(44.0)
Initial type of ET^a^		
Letrozole	57	(46.7)
Anastrozole	52	(42.6)
Tamoxifen	11	(9.0)
Exemestane	2	(1.6)
Median total follow-up in months (IQR)	47	(27–62)

Unless stated otherwise, numbers are shown as *n* (%)

^a^Categories may not sum to a total of 100% because of rounding

^b^Clinical node-positive disease was confirmed by imaging and pathology

*IQR* interquartile range, *No* number of, *T* tumour, *N* lymph node, *PR* progesterone receptor, *Her2* human epidermal growth factor receptor 2, *IDC* invasive ductal carcinoma, *ILC* invasive lobular carcinoma, *ET* endocrine therapy

The median period of follow-up was 47 months (IQR, 27–62 months). All 22 patients who started ET as NET to postpone surgery eventually did undergo surgery after a median period of 174 days (range 61–424). Nineteen of the 100 patients receiving ET as definitive treatment (*n* = 100) underwent invasive local treatment (surgery *n* = 14 and/or radiotherapy *n* = 6).

The number of patients actively treated with ET decreased from 122 patients at the start of the study period to 56 patients (46%) after 3 years. Over time, the proportion of these patients who visited their physician was 100, 88, 67, 56, and 73% during the 0–6, 6–12, 12–18, 18–24 months and 2–3 year periods, respectively. Among those who did visit the hospital, 85, 73, 61, 63, and 61% underwent tumour size measurements in the respective observation periods.

**Fig. 2:**
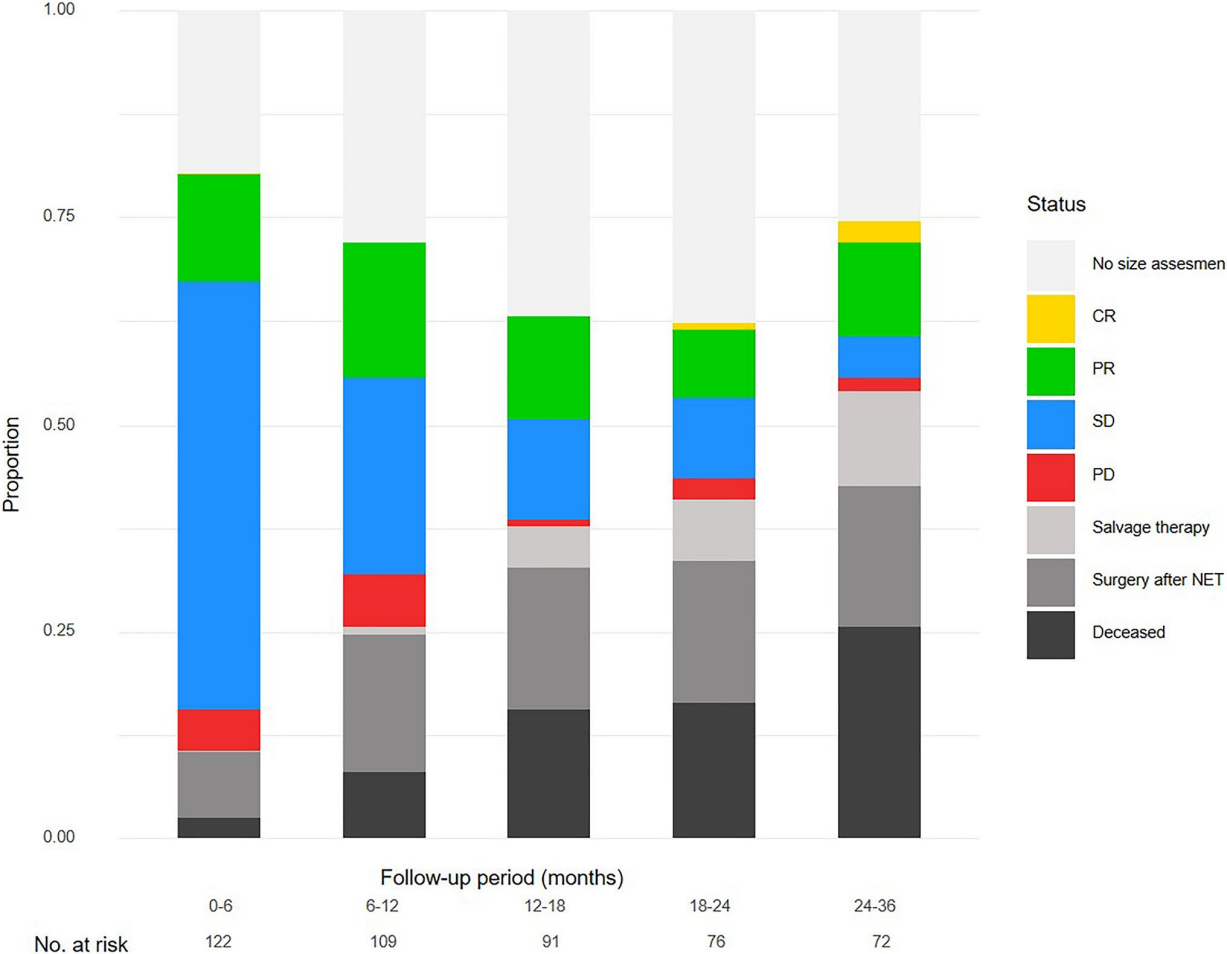
Frequency of tumour response to endocrine treatment over time in the context of overall survival and risk of invasive local treatment. Frequencies of ‘No size assessment’, ‘PD’, ‘SD’, ‘PR’ and ‘CR’ represent the status within the follow-up period. *CR* complete remission, *PR* partial remission, *PD* progressive disease, *SD* stable disease. Frequencies of ‘Deceased’, ‘Salvage therapy’, and ‘Surgery after NET’ categories represent terminal states and are presented cumulatively. The number at risk at the start of the follow-up period was defined as the number of patients who were alive and had not undergone salvage therapy or surgery after NET. No. at risk, Number at risk; ET, neo-adjuvant endocrine therapy

Figure 2 illustrates the proportions of all 122 patients in each observation period with the documented response assessment of that period alongside the cumulative risk over time of undergoing planned surgery, salvage therapy or death. At 3 years of follow-up, 25% of patients had died, 17% had undergone intended surgery following NET and 12% required salvage therapy. During the same observation period, 14% of all patients who had started ET were alive with objectivated clinical response: 14 patients with a partial response and 3 with a complete response. Stable disease decreased over time and was observed in only 5% of patients in the last observation period. Of the 63 patients who had at least one measured partial response, size reduction was confirmed by a subsequent measurement in 24 patients (38%). The overall response rate after 3 years was 14% for all 122 patients and 30% for the 56 patients who were still alive and had not undergone local treatment after 3 years.

By the end of the 5-year follow-up, 77 of the 122 patients had died and 11 had developed breast cancer metastases. For the entire cohort, the cumulative 5-year OS was 44% (Supplemental Fig. 1). For the 100 patients receiving ET as definitive treatment, the 5-year cumulative risk of undergoing invasive local treatment was 19% (Supplemental Fig. 2).

## Discussion

In this retrospective study in elderly patients who received ET as primary treatment, a clinical response was observed in substantial proportions of patients throughout the study period. While more than half of all patients either died or underwent invasive local treatment during the study period, the absolute number of patients with a clinical response stabilized over time.

To our knowledge, this is the first study to visualize the response to primary ET in elderly patients by presenting measured primary tumour response using RECIST cut-off volume reduction criteria. In the present study, thirty-percent of the patients who were still receiving ET at the end of the study period had an objectivated sustained clinical response. More than half of all patients were censored at that point because they had died or undergone invasive local treatment and were no longer receiving ET. A retrospective study by Osborn et al. [16] reporting results on 81 patients receiving ET showed progressive disease in 2, 5, and 15% of patients at 6 months, 2 and 6 years, respectively. In a retrospective study by Thomas et al. [17] involving 488 patients receiving ET, progressive disease was observed in 18% of patients after a median follow-up of 28 months. Wink et al. [13] found that 35% of patients eventually had progressed on ET, with a mean time to progression of 20 months.

The highest proportion of patients with stable disease or a clinical response to ET was observed in the first 6 months after treatment initiation, which aligns with findings from previous studies [18, 19]. From then on, the proportion of patients with stable disease gradually decreased, and in the last observation period (2–3 years), stable disease was rarely observed. Notably, complete clinical response was first observed after 1 year and slightly increased with longer treatment duration. Prolonged treatment may be necessary to achieve this outcome, which is supported by previous studies [19, 20]. A consistent proportion of all patients had a partial or complete response throughout the study period. These effects over time may hold clinical relevance, as it can help guide expectations regarding treatment efficacy. For clinicians considering ET, it is important to weigh this anticipated window of response against the patient’s overall prognosis and life expectancy.

The present study evaluates the clinical response in the context of cumulative risks of undergoing invasive local treatment or death. The 5-year OS in the present study was 44%, consistent with similar rates observed in other studies [7, 11, 14]. National statistics predict a median life expectancy of 6 years for 86-year-old Dutch women, which is slightly better than the OS in the study cohort and reflects the role of ET as a treatment option in patients with limited life expectancy. The selection of patients to whom ET was offered is exemplified in our study by their advanced age, high prevalence of comorbidities, and the small proportion of patients having metastatic disease at time of death. In the present study, approximately 15% of the deceased patients had metastases at the time of death, whereas in a previous study, conditions other than metastases or local disease progression were documented as the cause of death in 80% of deceased patients [14].

A systematic review by Morgan et al. [10] reported that many non-randomized studies suggest a significant benefit of surgery. As part of the Bridging the Age Gap in Breast Cancer Trial, Wyld et al. [21] more recently performed a prospective multicentre cohort study involving over 3,400 patients. After propensity-score matching, they found that surgical intervention is oncologically indeed superior to ET, but they also demonstrated that frail elderly women with a reduced life expectancy of less than 5 years may experience minimal survival benefit from surgery. The authors showed that these patients experienced significant short- and long-term deterioration in quality of life due to the operation [21]. An alternative option for these patients is wide local excision under local anaesthesia which has been shown as a viable option for elderly patients not willing or able to undergo surgery to avoid the drawbacks of general anaesthesia [22, 23].

In literature, there is no consensus on the age threshold for classifying patients as elderly, and it is often set at 70 years old [24]. However, it might be worth reconsidering this definition. Our study population had a mean age of 86 years, consistent with other studies on ET in elderly [16, 17, 21]. Given that the average life expectancy for females in Europe is 84 years [25], this suggests that the definition of ‘elderly’ might need to shift the threshold towards 80 years or older when referring to this select group of patients.

This study has many important limitations. As a retrospective study, patients were not evaluated using a standardised protocol, and response measurements were not performed consistently across all observation periods. This is illustrated by the substantial number of patients who did not visit their physician or did not undergo tumour size assessments in the respective observation periods. Moreover, measurements were not exclusively done with ultrasonography, and objectivated responses were not consistently confirmed. As a result, the outcomes are to be interpreted with caution. The exclusion of a substantial number of patients who received ET but were not actively monitored as they were commonly referred to their primary care or nursing home physicians is another important limitation. This introduces selection bias, as no information on tumour size was available for half of our study population. Furthermore, information regarding several biological characteristics, such as quantitative ER and Ki-67 expression, were not available and the malignancy grade for only a small proportion of patients. These factors could help predict the expected benefit of ET. An interesting area for further research is the use of the Ki-67 proliferation index [26]. Determining the Ki-67 index at the start of ET and after two weeks of treatment may, for example, allow for risk stratification and tailored follow-up schedules (e.g. low-risk vs. high-risk).

For frail and elderly patients diagnosed with ER-positive breast cancer, the option exists to forego surgery and initiate primary ET [27]. The observed results have to be interpreted with caution but do provide reassuring insight into the expected response over time in a vulnerable group of elderly patients for whom surgery is not the preferred treatment option. A substantial proportion of patients demonstrated a sustained clinical response to ET. However, patients with stable disease should be subject to close monitoring as timely local treatment should be discussed once their tumours progress, and efforts should be directed to distinguish those that will develop tumour progression or are likely to live beyond 3 years. By understanding the expected responses, better individualized patient care can be provided [28].

Based on these results, the sustained favourable clinical response to ET in a consistent proportion of patients suggests it may be a viable option for a selection of frail and elderly breast cancer patients with limited life expectancy. A standardized follow-up schedule with outpatient clinic visits supported by ultrasonography every 6 months is highly recommended for patients in both clinical and primary care settings. This will facilitate more consistent data collection and support better counselling of future patients and their physicians.

## Supplementary Information

Below is the link to the electronic supplementary material.

Supplementary file1 (DOCX 188 KB)

## Data Availability

No datasets were generated or analysed during the current study.
