# Supplementary material for: Evaluating clinical response to primary endocrine therapy in elderly breast cancer patients in routine practice
Source: Breast Cancer Res Treat. 2025 Sep 12;214(2):215–22. doi: 10.1007/s10549-025-07809-0 (PMC12464097; doi:10.1007/s10549-025-07809-0)

**Supplementary information**

**Supplemental Fig. 1** 10-year cumulative overall survival for the entire cohort (*n*=122).


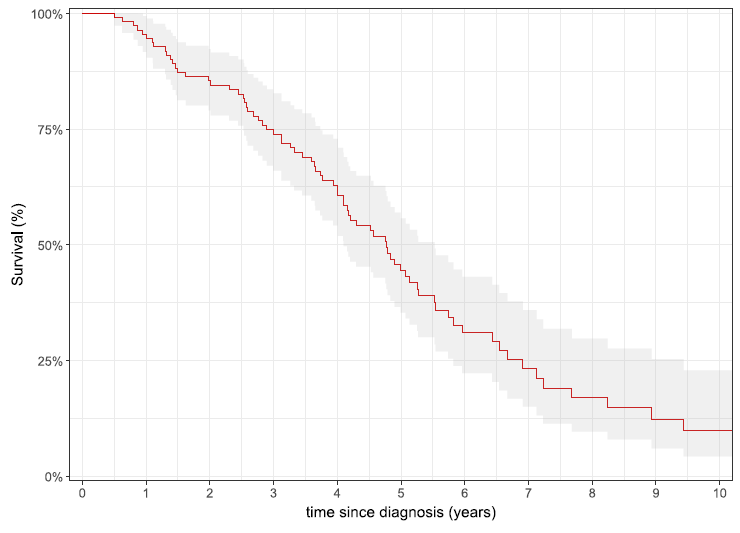


**Supplemental Fig. 2** 10-year cumulative risk of invasive local treatment (radiotherapy and/or surgery) for patients who received endocrine therapy as definitive treatment (*n*=100).


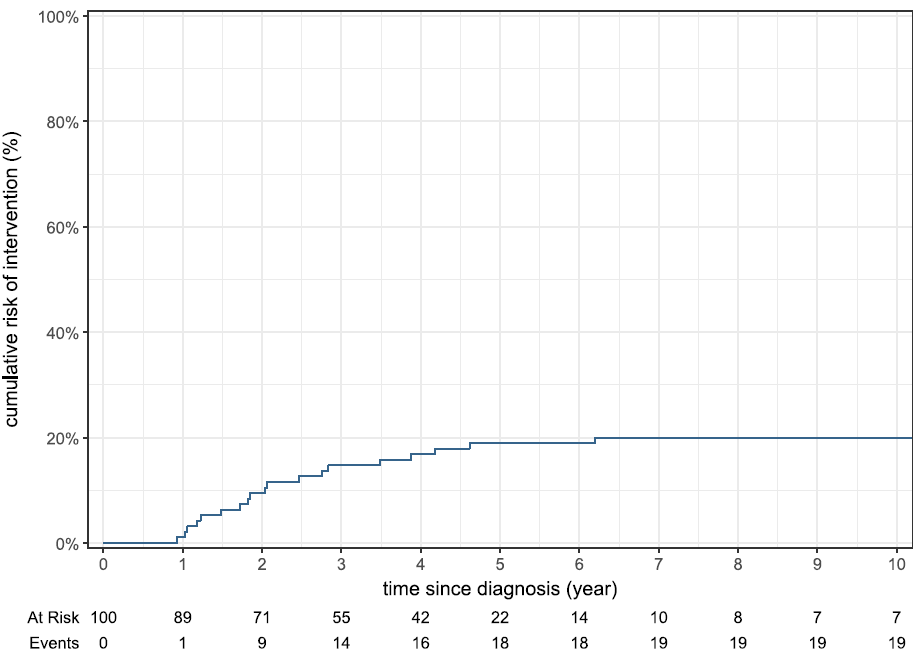

Supplement: Supplementary file 1 — Supplementary file1 (DOCX 188 KB) [file 10549_2025_7809_MOESM1_ESM.docx]
